# Does Religious Fasting Have a Protective Role against Metabolic Syndrome in Individuals Aged >50 Years?

**DOI:** 10.3390/nu15143215

**Published:** 2023-07-20

**Authors:** Anna Kokkinopoulou, Ioannis Pagkalos, Nikolaos E. Rodopaios, Alexandra-Aikaterini Koulouri, Eleni Vasara, Sousana K. Papadopoulou, Petros Skepastianos, Maria Hassapidou, Anthony G. Kafatos

**Affiliations:** 1 Department of Preventive Medicine and Nutrition Unit, School of Medicine, University of Crete, 71003 Crete, Greece; kokkinopoulouan@gmail.com (A.K.);; 2 Department of Nutritional Sciences and Dietetics, International Hellenic University, 57400 Thessaloniki, Greece; 3 Laboratory of Animal Physiology, Department of Zoology, School of Biology, Aristotle University of Thessaloniki, 54124 Thessaloniki, Greece; 4 Department of Medical Laboratory Studies, International Hellenic University, 57400 Thessaloniki, Greece

**Keywords:** nutrient intake, metabolic syndrome, obesity, religious fasting, Christian Orthodox Church fasting, Mediterranean Diet

## Abstract

Objective: The Christian Orthodox Church (COC) fasting is a periodic vegetarian-type diet lasting 180–200 days annually in which seafood and snails are allowed on all fasting days. Our aim was to investigate the effects of COC fasting on metabolic syndrome (MetS) in adults >50 years. Methods: One hundred seventy-six individuals participated in the study (mean age 59.7 ± 6.0 years). Eighty-nine had been following the COC fasting regime since their childhood and eighty-seven were non-fasters. Anthropometric measurements, blood samples, and nutrient intake data were collected at a scheduled appointment, during a non-fasting period. Results: Fasters had significantly higher hip circumference (102 ± 8.5 vs. 98.8 ± 7.5 cm, *p* = 0.009), low-density lipoprotein (LDL) cholesterol (136 ± 73 vs. 115 ± 51 mg/dL, *p* = 0.033), and folic acid levels (6.0 ± 4.0 vs. 3.0 ± 1.2 ng/mL, *p* = 0.018), as well as lower systolic blood pressure (SBP) (131 ± 13 vs. 136 ± 14 mmHg, *p* = 0.045), diastolic blood pressure (DBP) (80 ± 8 vs. 83 ± 7 mmHg, *p* = 0.007), glucose (87 ± 16 vs. 93 ± 25 mg/dL, *p* = 0.039), and triglycerides (143 ± 94 vs. 175 ± 84 mg/dL, *p* = 0.040). Fasters had significantly less intake of calories (1493.65 ± 363.74 vs. 1614.65 ± 426.28 kcal, *p* = 0.044) and fat (81.17 ± 25.47 vs. 90.74 ± 24.75 g, *p* = 0.012), whereas they also consumed significantly less vitamin A-retinol, vitamin B2, vitamin B12, vitamin E, folic acid, pantothenic acid, calcium, phosphorus, zinc, and significantly more vitamin C (*p* < 0.005 for all comparisons). BP was significantly higher in non-fasters (44.8 vs. 22.5%, *p* = 0.002), whereas MetS was more frequently present in non-fasters vs. fasters, with a marginal level of significance (35.6 vs. 22.5%, *p* = 0.055). Conclusions: COC fasting may affect beneficially MetS prevalence in individuals >50 years, although future research is needed before reaching definite conclusions.

## 1. Introduction

Metabolic syndrome (MetS) is a cluster of disorders, contributing to the risk of developing cardiovascular diseases, type 2 diabetes, and other health problems [1]. According to the joint criteria of the International Diabetes Federation Task Force on Epidemiology and Prevention, the National Heart, Lung, and Blood Institute, the American Heart Association, the World Heart Federation, the International Atherosclerosis Society, and the International Association for the Study of Obesity, MetS is diagnosed with the presence of three or more of the following criteria: (a) elevated waist circumference (≥102 cm and ≥88 cm for males and females, respectively), (b) elevated fasting blood glucose (≥100 mg/dL), (c) elevated triglycerides (≥150 mg/dL), (d) decreased high-density lipoprotein (HDL) cholesterol (<40 and <50 mg/dL for males and females, respectively), and (e) elevated systolic blood pressure (SBP) and diastolic blood pressure (DBP) (≥130/85 mmHg) [2,3].

According to the joint criteria, MetS prevalence in Greek populations varied from 19.8% in the Attica study [4] to 23.6% in the MetS-Greece Multicentre Study [5]. MetS is influenced by various factors, including dietary habits, genetics, and a sedentary lifestyle, therefore, its prevention and treatment combine following a healthier diet, losing body weight, and being physically active [6,7]. 

The Mediterranean Diet is a plant-based dietary pattern that is characterized by increased consumption of wholegrain cereals, legumes, fruits, vegetables, nuts, pulses, seafood, and olive oil [8,9] and has its roots in the traditional Cretan Mediterranean Diet [8,10]. The Christian Orthodox Church (COC) fasting dietary pattern has been the origin and the main characteristic of the diet of Crete and Greece for over 2000 years [11]. The COC fasting dietary pattern is an interchange of a vegetarian diet with fish, seafood, and snails (as some of the protein sources) during fasting periods, to a mixed diet that also includes meat, dairy products, and eggs. Olive oil is usually allowed on certain days, even during major fasting periods. Fasting in the COC lasts 180–200 days annually and takes place in four major periods (Nativity, Lent, Assumption, and Saint Apostles fasting), in addition to almost every Wednesday and Friday and three other daily feasts (5 January, 29 August, and 14 September) [12]. 

The COC fasting regime is rooted in the tradition of penance, prayer and spiritual reflection; therefore, some regional and/or individual variations might exist, but the basic principles remain the same. Also, a healthier way of living is promoted in general through the COC recommendations, with the abstinence from alcohol and smoke [13,14]. It is important to mention that abstinence from alcohol includes wine, beer, and other alcoholic beverages [12].

The impact of COC fasting on human health has been a subject of growing interest among researchers during the last decades. Evidence has shown possible preventive effects of religious fasting on obesity, cardiometabolic risk factors, and type 2 diabetes mellitus [12,15,16,17]. Of note, according to a recent review, COC fasting is highlighted as a sustainable diet, as its recommendations may have a positive impact on planetary health [18]. 

The aim of the present study was to study a Greek COC fasting population aged >50 years, with respect to the prevalence of cardiometabolic risk factors and nutrient intake.

## 2. Materials and Methods

### 2.1. Population

This was a cross-sectional study that took place at the Department of Nutritional Sciences and Dietetics, in Thessaloniki, Greece, and it was designed to investigate the effects of COC fasting on health. Volunteers for the study were recruited via a call for participants that was disseminated in public Universities, monasteries, and churches in Thessaloniki, the second-largest city in Greece. Ethical approval of the study was given by the Bioethics Committee of the Alexander Technological Educational Institute.

The purpose and the protocol of the study were explained in detail to individuals who expressed their will to participate. All participants gave written informed consent and were then asked to fill out a closed-ended questionnaire for their diet habits in order to designate their participation. Individuals who could participate in the study as fasters had to declare their adherence to the COC fasting recommendation throughout the fasting calendar, that is spread in 180–200 days in the year, since their childhood or at least the last twelve consecutive years. According to the literature, people who follow this diet pattern from their childhood tend to follow it throughout their life, as it is part of their culture and religion [13]. Individuals who did not follow the same dietary pattern and did not abstain from any food item (e.g., lactose, wheat, etc.) due to medical and/or lifestyle reasons were included in the study as non-fasters. All participants were free to withdraw from the study with no consequences. 

Exclusion criteria were (a) not being >50 years, (b) not providing written informed consent, (c) not participating at the scheduled appointment to collect measurements, (d) having non-communicable diseases (NCDs), such as heart disease, cancer, and other, (e) having food allergies. Overall, there were 176 individuals (62 men and 114 women) aged 51 to 77.5 years (mean age 59.7 ± 6.0 years) participating in the study. Eighty-nine individuals (31 men and 58 women) aged 51 to 77.5 years (mean age 60.0 ± 6.0 years), fasted regularly according to the fasting periods of COC, for a mean period of 34.4 ± 12.3 years, starting since their childhood or for the last twelve years. Another group of eighty-seven individuals (31 men and 56 women) aged 51 to 77.3 years (mean age 59.5 ± 6.0 years) were non-fasters. 

### 2.2. Variables Collected

The study protocol was based on a systematic method used by health care professionals and nutritionists to assess, diagnose, treat, evaluate, and monitor people, that is called the nutrition care process (NCP) model approach [19]. A trained registered nutritionist performed all measurements and collected all data, apart from biochemical that were collected with the help of a registered nurse. All participants were asked to complete a validated Greek population questionnaire with yes/no, open-ended, and closed-ended questions, in relation to educational level, marital status, smoke status, sleeping habits, physical activity status, and diet supplement use among other lifestyle habits [20].

According to the NCP, anthropometrics were collected in order to reveal the nutritional status, growth, and health of individuals. Body height, body weight, waist, and hip circumference, and body composition were measured, with participants fasting after midnight on the day before the appointment, barefoot and in minimal clothing. Body height was measured with the HR001 TANITA stadiometer (TANITA, Leicester, UK) to the nearest 0.5 cm, following the Frankfort Plane position. Body weight was measured with the UM075 TANITA digital scale (TANITA, Amsterdam, The Netherlands) to the nearest 0.1 kg. After collecting these two measurements, body mass index (BMI) was calculated to be used for further nutritional status classification of participants. Waist and hip circumference were measured with the use of a SECA 201 body girth tape (SECA, Hamburg, Germany) to the nearest 0.1 cm. Similarly, with the use of the two aforementioned measurements, waist-to-hip ratio (WHR) was calculated for further classification of abdominal obesity. Body fat, fat mass, muscle mass, and total body water were measured via the bioelectrical impedance analysis (BIA) method with the use of BODYSTAT 1500 bioimpedance analyser (BODYSTAT, Warwickshire, UK). Last, blood pressure (BP) was measured with the Omron BP monitor (Omron, Hoffman Estates, IL, USA), and the researcher recorded both systolic BP (SBP) and diastolic BP (DBP) values.

Six mL of venous blood was collected with all analysis performed at a certified lab, and personal and family history data were collected through the validated questionnaire [20]. Also, a combination of methods was used to collect comprehensive dietary data. Hence, three 24 h diet recalls, and a food frequency questionnaire (FFQ) was used to collect detailed information about all foods and beverages consumed during a week and a month, respectively. For the accuracy of portions, we used a combination of plastic/rubber food replicas (NASCO, Fort Atkinson, WI, USA), household cups and plates, and a food atlas [21]. For the analysis of the 24 h diet recalls we used the Food Processor nutrition analysis software (v.11.7) (ESHA, Salem, OR, USA), in which Greek food recipes were added in its database from the Food Composition Tables and Composition of Greek Food and Dishes [22]. What is more, a validated questionnaire to assess nutritional behavior of participants was used, with questions for breakfast consumption, mindful eating, eating and cooking habits, and supplement use among other [20].

### 2.3. Statistical Analysis 

The SPSS statistical software v.21 (IBM, New York, NY, USA) was used for all presented data analyses. Continuous data were checked for normality of distribution with the Kolmogorov–Smirnov test. Continuous data are presented in the following tables as Means with Standard Deviation (SD) and categorical variables as Frequencies (%). The Chi-Squared test was used to test for differences among categorical variables, while the Student’s *T*-test and One Way Analysis of Variance (ANOVA) for differences in continuous variables. Statistical significance was set at a *p*-value of 0.05.

## 3. Results

A total of 62 men and 114 women participated in the study and had a mean age of 59.7 ± 6.0 years, a mean weight of 77.4 ± 13.4 kg, a mean BMI of 28.43 ± 4.11 kg/m^2^, a mean waist circumference of 95.2 ± 11.6 cm, a mean hip circumference of 100.4 ± 8.2 cm, and a mean waist-to-hip ratio of 0.95 ± 0.1. Eighty-nine fasters had a mean age of 60 ± 6 years, a mean weight of 78 ± 13.4 kg, a mean BMI of 28.7 ± 4.2 kg/m2, a mean waist circumference of 95.8 ± 12.1 cm, a mean hip circumference of 102 ± 8.5 cm, and a mean waist-to-hip ratio of 0.94 ± 0.1. Eighty-seven non-fasters had a mean age of 59.5 ± 6 years, with a mean weight of 77 ± 13.4 kg, a mean BMI of 28.1 ± 4.0 kg/m^2^, a mean waist circumference of 94.6 ± 11.1 cm, a mean hip circumference of 98.8 ± 7.4 cm, and a mean waist-to-hip ratio of 0.9 ± 0.1 (details can be seen in Table 1).

No differences were observed in anthropometric variables among fasters and non-fasters, except for hip circumference, with fasters having higher mean values compared with non-fasters (102.03 ± 8.57 vs. 98.83 ± 7.42 cm, *p* = 0.009), as well as for SBP (131 ± 13 vs. 136 ± 14 mmHg, *p* = 0.045), and DBP (80 ± 8 vs. 83 ± 7 mmHg, *p* = 0.007) both being significantly lower in fasters (see Table 1).

More than half of the sample had tertiary education and above (*n* = 89, 50.6%), most were married (*n* = 148, 84.1%), and never smoked (*n* = 142, 80.7%). Among smokers, only a few (*n* = 17, 9.7%) smoked more than 10 cigarettes per day. When analysis was carried out based on diet status, there was a significant relationship between diet status and education level (*p* = 0.001) as well as family status (*p* = 0.031). In more detail, 52.8% of fasters (*n* = 47) had an education level from the tertiary level and above compared with 48.2% of non-fasters (*n* = 42, *p* = 0.001). Also, 87.4% of non-fasters were married vs. 80.9% of fasters (*n* = 76 and *n* = 72, respectively, *p* = 0.031). No significant differences were found based on gender (*p* = 0.61), smoking status (*p* = 0.80), and BMI (*p* = 0.08). Also, no significant difference was found in table salt use, with 69.7% of fasters and 74.7% of non-fasters not using more table salt, apart from cooking, in all their meals (*p* = 0.45). Results from the comparative analysis for the diet groups (fasters vs. non-fasters) are included in Table 2. 

Referring to blood lipids, fasters had significantly lower mean triglycerides (143 ± 94 vs. 175 ± 84 mg/dL, *p* = 0.040) and higher mean LDL cholesterol (136 ± 73 vs. 115 ± 51 mg/dL, *p* = 0.033), while HDL and total cholesterol did not differ significantly. Furthermore, fasters had significantly lower mean values in fasting glucose (87 ± 16 vs. 93 ± 25 mg/dL, *p* = 0.039), non-significantly lower levels of Vitamin D and Vitamin B12 (*p* = 0.20 and *p* = 0.53, respectively), and non-significantly higher levels of iron (*p* = 0.86), while they had significantly higher levels of folic acid (*p* = 0.018) (Table 3).

With respect to the five different MetS components, significantly more non-fasters had elevated BP compared with non-fasters (*n* = 39 vs. 20 fasters, *p* = 0.002). Overall, MetS prevalence was more common in non-fasters vs. fasters with a marginal level of significance (*n* = 20 fasters vs. 31 non-fasters, *p* = 0.055) (Table 4). When the analysis was carried out based on gender, significantly more women had elevated waist circumference (*p* = 0.001) and high levels of HDL cholesterol (*p* = 0.017), while more men had increased levels of BP (*p* = 0.001). Although there are significant differences in men vs. women, MetS presence was not significantly different (*p* = 0.72).

In relation to MetS components, significantly more non-fasters had ≥ 3 variables compared with fasters (*p* = 0.034). In more detail, three components were found in 14.6% of fasters vs. 17.2% of non-fasters (*n* = 13 vs. *n* = 15, respectively), four components in 6.7% of fasters vs. 10.3% of non-fasters (*n* = 6 vs. *n* = 9, respectively), and five components in 1.1% of fasters vs. 8% of non-fasters (*n* = 1 vs. *n* = 7, respectively).

Diet status, gender, education level, marital status, smoking status, number of cigarettes/days, physical activity status, free time workout, and total energy intake were used in the general linear model to identify any associations with MetS. The final adjusted model included gender (*p* = 0.027), number of cigarettes/day (*p* = 0.003), physical activity status (*p* = 0.025), free time workout (*p* = 0.005), and total energy consumed (*p* = 0.001). It was shown that the MetS prevalence was lowered in women, and in participants with higher physical activity and increased free time workout. On the other hand, it was increased with smoking and higher energy intake.

With reference to the three 24 h dietary recalls, fasters consumed significantly less calories (1493.65 ± 363.4 vs. 1614.65 ± 426.28 kcals, *p* = 0.044) and less total fat (81.17 ± 25.47 vs. 90.74 ± 24.75 g, *p* = 0.012), while total protein and carbohydrate intake was not significantly different (50.69 ± 17.45 vs. 54.42 ± 16.99 g, *p* = 0.15, and 150.44 ± 48.82 vs. 154.75 ± 57.09 g, *p* = 0.58, respectively). Fasters had significantly lower mean intake of vitamin A—carotenoid (216.72 ± 26 vs. 294.8 ± 53.22 μg, *p* = 0.032), vitamin B2 (1.1 ± 0.4 vs. 1.3 ± 0.6 μg, *p* = 0.010), vitamin B12 (2.5 ± 0.3 vs. 4.1 ± 0.6 μg, *p* = 0.012), vitamin E (6.3 ± 3 vs. 9 ± 3.4 μg, *p* = 0.002), folic acid (176.7 ± 82.5 vs. 214.15 ± 110 mg, *p* = 0.012), and pantothenic acid (2.1 ± 0.9 vs. 2.6 ± 1 mg, *p* = 0.017), while higher mean intake of vitamin C (249.8 ± 37.1 vs. 132.3 ± 22.1 μg, *p* = 0.008). Regarding mineral intake, fasters had significantly lower mean intake of calcium (578.12 ± 272.68 vs. 691.70 ± 331.6 mg, *p* = 0.014), phosphorus (775.6 ± 274.4 vs. 892.9 ± 375.6 mg, *p* = 0.019), and zinc (6.41 ± 3 vs. 7.73 ± 3.5, *p* = 0.008), and the mean intake of the rest of the minerals were non-significantly different between fasters and non-fasters. All aforementioned differences can be seen in the following Table 5.

## 4. Discussion 

To the best of our knowledge, little research has focused on the effects of COC fasting and nutrient intake on MetS prevalence, during a non-fasting period. According to a review, adherence to the COC fasting recommendations throughout the year, i.e., for 180–200 days, could be beneficial for lipid profiles; however, the evidence is still limited [2,12].

In this cross-sectional study, fasters consumed significantly less energy and total fat compared with non-fasters. Despite these differences, the prevalence of overweight and obesity was similar in both fasters and non-fasters (80.9% and 74.7%, respectively). This could be due to self-reporting of food intake, as well as to the low physical activity reported by both fasters (41.6%) and non-fasters (46%).

Our study showed that fasters had significantly lower SBP and DBP (*p* = 0.045 and *p* = 0.007, respectively) when compared to non-fasters. In another Greek COC fasting population, after the Easter, Christmas, and Assumption fasting periods, mean SBP was decreased in fasters (*n* = 38) compared with non-fasters (*n* = 39). In the same study individuals who had elevated BP, during all periods, had older age and increased BMI [23]. Furthermore, after the Easter fasting period in an Egyptian population (*n* = 49), COC religious fasting reduced SBP in individuals with and without type 2 diabetes mellitus, as well as SBP and DBP in those with hypertension [24]. It is worth mentioning that, according to recent reviews, plant-based, and/or vegetarian diets are associated with lowering BP levels when compared to omnivorous diets [25,26]. The positive benefits of plant-based diets in lowering BP levels are further revealed in the European Prospective Investigation into Cancer and Nutrition-Oxford study, where it was shown that vegetarian and vegan participants (*n* = 31,546) had the lowest prevalence of BP when compared to meat eaters (*n* = 33,883) [27,28]. Similarly, in the Adventist Health Study-2 vegetarians (*n* = 302) had lower SBP and DBP compared with omnivores (*n* = 198) [29].

Fasters had non-significantly lower levels of HDL and total cholesterol. In another study, COC fasters from Greek Monasteries had significantly lower values of HDL, LDL, and total cholesterol after the Christmas fasting period [24]. Similar results were reported in another Greek COC population, which found significantly lower levels of total cholesterol in fasters (*n* = 60) after the Christmas fasting period compared with non-fasters (*n* = 60), but these low levels were not maintained in the following non-fasting period [30,31]. Furthermore, in a population of sixty fasters with and without dyslipidemia, a significant reduction of HDL, LDL and total cholesterol levels was reported after seven consecutive weeks of COC fasting [32]. 

In this cross-sectional study, fasters had significantly lower levels of triglycerides. Controversial data exist in the literature. In a study conducted in Egypt by Elsayed and colleagues, triglyceride levels were reduced in 49 fasters with type 2 diabetes mellitus after a 48 days fasting period. However, in the same study, it was shown that glucose levels were not affected [24]. In another study, 37 fasters in Greece lowered their triglyceride levels after a 40-day fasting period [31]. On the other hand, in 99 people who followed a fasting period of 48 days, triglyceride levels did not change significantly, but glucose was significantly lowered [33]. Similarly, glucose levels remained the same in 36 COC fasters from Egypt that followed 43 days of fasting before Christmas [34]. According to the literature, plant-based diets can significantly improve glucose levels in individuals with overweight and/or obesity after interventions lasting from 12 weeks [35], 16 weeks [36], and up to 3 months [37].

In our study, significantly lower intakes of vitamins A, B2, B12, E, folic acid, pantothenic acid, calcium, phosphorus, zinc, and higher intakes of vitamin C were reported. These significant differences can be explained by the refraining from red and white meat, dairy products and eggs, and the increased consumption of fruits and vegetables that fasters exhibit. The study was conducted during a non-fasting period, although, as explained, COC fasting recommendations are followed every Wednesday and Friday throughout the year. In agreement to our study, significantly lower intakes of vitamin B12 and D, as well as calcium and zinc were reported from a seven-day weighed food record from 36 Egyptian COC fasters [34]. Significantly lower intake of calcium and vitamins A, B2, and E, and an adequate intake of vitamin C were found in a prospective study in Greece including 100 fasters, as showed by a two-day weighted food record [38,39]. Lastly, in a case–control study with a three-day weighed food record, 60 COC fasters consumed significantly less calcium, sodium, phosphorus, vitamins B1, B3, and B6 compared with 60 non-fasters [40,41]. Furthermore, a significantly increased intake of vitamin C was reported as a result of a 40-day fasting period before Christmas holidays in 35 COC Greek fasters vs. 24 non-fasters [41]. Of note, plant-based diets have been linked to reduced intake of vitamins B12, D, calcium, zinc, and iodine [42].

In our study, more non-fasters had MetS with a marginal level of significance when compared with fasters. This is of great importance, as it reveals a possible protective effect of the COC fasting diet, although more studies with higher numbers of participants are needed and with a longer follow-up. It is worth mentioning that plant-based diets can reduce the prevalence of MetS components, as well as risk factors for CVD [42,43]. 

In the present study, fasters seemed to follow a healthier way of lifestyle, with 97.7% of them being non-smokers (having never smoked or quit smoking), compared to 34.5% of non-fasters. This is in agreement with a Greek study that revealed that COC fasting was associated with positive health-related behaviors, such as abstinence from smoking and alcohol [13].

The present study has some limitations, starting with the fact that the majority of participants were women, and we are unsure about the effects of gender on the outcomes. Also, blood chemistry parameters and blood pressure were only taken once during the scheduled appointment, therefore, the biological significance of these changes is not known at this time and requires further investigation. Epidemiological studies have a well-known drawback that is the misreporting of dietary intake and that might lead to false conclusions. However, in order to eliminate this disadvantage and collect reliable dietary intake data, food replicas, and food atlas, with the supervision of a registered nutritionist, were used in the present study. Although the sample size of this study seems small (i.e., 89 fasters and 87 non-fasters), the study refers to a non-fasting period, revealing the habits of their everyday life. In this context, the COC fasting regime could be easily adopted by individuals who want to follow a plant-based diet and according to a recent review, COC fasting is considered a sustainable diet that could be used by public health authorities to ensure a sustainable healthy planet and promote healthy living [18].

## 5. Conclusions

The findings of the present study suggest that MetS prevalence was more common in non-fasters vs. fasters, with a marginal level of significance, in adults >50 years old. Fasters had significantly lower SBP, DBP, fasting glucose, and triglycerides levels, as well as significantly elevated HDL cholesterol vs. non-fasters. The intake of certain vitamins and nutrients was different between fasters and non-fasters.

By investigating in detail, the COC fasting dietary pattern and the associations with blood lipids and the lifestyle habits of individuals, public health authorities, and nutritionists would shed light on the actions needed to be addressed to tackle MetS. Taking into consideration the significant impact MetS has on public health, future studies are needed before establishing definite conclusions. 

## Figures and Tables

**Table 1:** Anthropometric variables.

Variable	Fasters (*n* = 89)	Non-Fasters (*n* = 87)	*p*-Value
	Mean ± SD	Mean ± SD	
**Weight (kg)**	77.8 ± 13.4	77 ± 13	0.67
**Height (m)**	1.64 ± 0.08	1.65 ± 0.08	0.52
**BMI (kg/m^2^)**	28.7 ± 4.2	28.1 ± 4	0.31
**Body fat (%)**	34.7 ± 8.7	35.4 ± 6.6	0.57
**Body fat (kg)**	27.3 ± 9.2	27.3 ± 7.3	0.98
**Fat free mass (kg)**	50.4 ± 9.6	49.5 ± 9.8	0.55
**Waist circumference (cm)**	95.7 ± 12.1	94.6 ± 11.1	0.52
**Hip circumference (cm)**	102 ± 8.5	98.8 ± 7.5	0.009
**WHR**	0.94 ± 0.1	0.95 ± 0.1	0.24
**SBP (mmHg)**	131 ± 13	136 ± 14	0.045
**DBP (mmHg)**	80 ± 8	83 ± 7	0.007
**Pulses (per minute)**	69 ± 9	70 ± 9	0.84

SD: Standard Deviation, BMI: Body Mass Index, WHR: Waist to Hip Ratio, SBP: Systolic Blood Pressure, DBP: Diastolic Blood Pressure.

**Table 2:** Demographic variables in fasters and non-fasters.

Variable	Fasters (*n* = 89)	Non-Fasters (*n* = 87)	*p*-Value
	*n* (%)	*n* (%)	
**Sex**			0.61
Male	31 (34.8)	31 (35.6)	
Female	58 (65.2)	56 (64.4)	
**Education level**			0.001
Primary education	9 (10.1)	15 (17.2)	
Secondary education	33 (37.1)	30 (34.5)	
Tertiary education	34 (38.2)	35 (40.2)	
Master’s/Doctoral	13 (14.6)	7 (8)	
**Marital status**			0.031
Single	13 (14.6)	1 (1.1)	
Married/Living together	72 (80.9)	76 (87.4)	
Divorced	4 (4.5)	5 (5.7)	
Widowed	-	5 (5.7)	
**Smoking status**			0.80
Yes	2 (2.2)	57 (65.5)	
No—never	85 (95.5)	30 (34.5)	
No—quit smoking	2 (2.2)	-	
**BMI status**			0.08
Normal weight	17 (19.1)	22 (25.3)	
Overweight	39 (43.8)	36 (41.4)	
Obesity	33 (37.1)	29 (33.3)	
**Physical activity level**			0.12
Extremely low	4 (4.5)	15 (17.2)	
Low	33 (37.1)	28.7 (28.7)	
Moderate	39 (43.8)	36 (41.4)	
High	12 (13.5)	11 (12.6)	
Extremely high	1 (1.1)		
**Free time workout**			0.28
Yes	64 (71.9)	56 (64.4)	
No	25 (28.1)	31 (35.6)	
**Salt use**			0.45
Yes	62 (69.7)	65 (74.7)	
No	27 (30.3)	22 (25.3)	

**Table 3:** Biochemical variables in fasters and non-fasters.

Variable	Fasters (*n* = 89)	Non-Fasters (*n* = 87)	*p*-Value
	Mean ± SD	Mean ± SD	
**Glucose (mg/dL)**	87 ± 16	93 ± 25	0.039
**Triglycerides (mg/dL)**	143 ± 94	175 ± 84	0.040
**HDL cholesterol (mg/dL)**	52 ± 17	54 ± 17	0.50
**LDL cholesterol (mg/dL)**	136 ± 73	115 ± 51	0.033
**Total cholesterol (mg/dL)**	210 ± 48	219 ± 50	0.24
**Vitamin D (ng/mL)**	17 ± 6	18 ± 6	0.20
**Vitamin B12 (pg/mL)**	300 ± 158	315 ± 163	0.53
**Iron (mg/dL)**	98 ± 32	97 ± 40	0.86
**Folic acid (ng/mL)**	6 ± 4	3 ± 1	0.018

SD: Standard Deviation, HDL: High-Density Lipoprotein, LDL: Low-Density Lipoprotein.

**Table 4:** Prevalence of MetS and its components in fasters and non-fasters.

Variable	Fasters (*n* = 89)	Non-Fasters (*n* = 87)	*p*-Value
	*n* (%)	*n* (%)	
**WC > 102 cm for men or >88 cm for women**	52 (58.4)	49 (56.3)	0.77
**FBG ≥ 100 mg/dL**	11 (12.4)	20 (23.0)	0.83
**HDL cholesterol < 40 mg/dL for men or <50 mg/dL for women**	31 (34.8)	29 (33.3)	0.07
**TRG ≥ 150 mg/dL**	32 (36)	43 (49.4)	0.06
**BP ≥ 130/85 mmHg**	20 (22.5)	39 (44.8)	0.002
**MetS prevalence**	20 (22.5)	31 (35.6)	0.055

WC: Waist Circumference, FBG: Fasting Blood Glucose, HDL: High-Density Lipoprotein, TRG: Triglycerides, BP: Blood Pressure, and MetS: Metabolic Syndrome.

**Table 5:** Nutrient intake in fasters and non-fasters.

Variable	Fasters (*n* = 89)	Non-Fasters (*n* = 87)	*p*-Value
	Mean ± SD	Mean ± SD	
**Energy (kcal)**	1493.65 ± 363.74	1614.65 ± 426.28	0.044
**Protein (g)**	50.69 ± 17.45	54.42 ± 16.99	0.15
**Carbohydrates (g)**	150.44 ± 48.82	154.78 ± 57.09	0.58
**Fat (g)**	81.17 ± 25.47	90.74 ± 24.75	0.012
**Vit A—Carotenoid (μg)**	254.8 ± 29.2	294.8 ± 53.22	0.50
**Vit A—Retinol (μg)**	216.72 ± 26	379.65 ± 71.2	0.032
**Vit A—Beta carotene (μg)**	2208.4 ± 177.3	2335.2 ± 272.8	0.69
**Vit B1 (μg)**	1.2 ± 0.1	1.1 ± 0.4	0.44
**Vit B2 (μg)**	1.1 ± 0.4	1.3 ± 0.6	0.010
**Vit B3 (μg)**	9 ± 5.4	10 ± 5	0.21
**Vit B6 (μg)**	1.4 ± 0.2	1.3 ± 0.1	0.76
**Vit B12 (μg)**	2.5 ± 0.3	4.1 ± 0.6	0.012
**Vit C (μg)**	249.8 ± 37.1	132.3 ± 22.1	0.008
**Vit D (μg)**	2.04 ± 0.3	2.30 ± 0.3	0.52
**Vit E (μg)**	6.3 ± 3	9 ± 3.4	0.002
**Folic acid (mg)**	176.7 ± 82.5	214.15 ± 110	0.012
**Pantothenic acid (mg)**	2.1 ± 0.9	2.6 ± 1	0.017
**Calcium (mg)**	578.12 ± 272.68	691.70 ± 331.6	0.014
**Copper (mg)**	0.64 ± 0.03	0.74 ± 0.04	0.07
**Iron (mg)**	9.24 ± 3.42	10.5 ± 5.44	0.06
**Magnesium (mg)**	169.3 ± 55.6	179.9 ± 67.7	0.25
**Manganese (mg)**	1.21 ± 0.8	1.37 ± 0.8	0.22
**Phosphorus (mg)**	775.6 ± 274.4	892.9 ± 375.6	0.019
**Potassium (mg)**	2018.3 ± 576.3	1937.1 ± 708.1	0.40
**Selenium (mg)**	46.22 ± 25.44	51.28 ± 27.88	0.21
**Sodium (mg)**	1691.1 ± 192.2	1748.9 ± 118.4	0.78
**Zinc (mg)**	6.41 ± 3	7.73 ± 3.5	0.008

## Data Availability

The raw data supporting the conclusions of this study are available from the corresponding author upon request.
